# Sustainable Bio-Based Polymers: Towards a Circular Bioeconomy

**DOI:** 10.3390/polym14010022

**Published:** 2021-12-22

**Authors:** Susana Guzman-Puyol, José J. Benítez, José A. Heredia-Guerrero

**Affiliations:** 1 Instituto de Hortofruticultura Subtropical y Mediterránea “La Mayora”, Universidad de Málaga-Consejo Superior de Investigaciones Científicas (IHSM, UMA-CSIC), Bulevar Louis Pasteur 49, 29010 Malaga, Spain; susana.guzman@csic.es; 2 Instituto de Ciencia de Materiales de Sevilla, Centro Mixto CSIC-Universidad de Sevilla, Calle Américo Vespucio 49, Isla de la Cartuja, 41092 Seville, Spain; benitez@icmse.csic.es

The valorization of biomass from different renewable resources (i.e., agriculture, food, forestry, fishery, etc.) by green processes to produce multifunctional polymers with potential industrial applications is a smart strategy to substitute non-biodegradable petroleum-based plastics [1,2,3,4,5,6,7,8,9,10,11,12]. This allows a change of paradigm from a linear economy (“take, make, and dispose”) to a circular bioeconomy where typical plant and animal residues littered as wastes have a second chance and can be re-integrated into the productive cycle, thus reducing their environmental impact. In this context, the works published in this Special Issue have addressed such a topic from different perspectives, including bio-based alternatives to petroleum-based polymers [3,8,9,12], bioplastics for packaging [2,4,10] and other industrial uses [6,7], and sustainable food waste management and biorefinery [1,5,11].

In summary, ten papers about the fabrication, characterization, and potential uses of bio-based polymers and composites have been published in this Special Issue [1,2,5,6,7,8,9,10,11,12]. It also contains two reviews about sustainable substitutes of phenol-formaldehyde resins [3] and synthetic and non-synthetic materials for packaging and textile uses [4].

Micó-Vicent et al. prepared new anthocyanin hybrid nanopigments and fabricated new polyester-based biocomposites with them [1]. For this, they extracted anthocyanins from pomegranate wastes and incorporated them to calcined hydrotalcite (HT) and montmorillonite (MMT) nanoclays. By changing the pH, a wide range of colors were obtained for MMT-based nanopigments, while HT buffered the pH and no different colors were achieved. Both nanopigments were included in polyester matrices to form bionanocomposites. MMT-based bionanocomposites were better than HT ones in terms of thermal stability, final color, and mechanical properties. The life cycle analysis (LCA) of the whole process evidenced its sustainability.

Aziman et al. reported an investigation about the incorporation of Biomaster-silver (BS) particles into poly(butylene succinate) (PBS) and PBS/tapioca starch (TPS) polymer matrices as food packaging films [2]. BS particles were well distributed in both polymer matrices and interacted preferentially with the PBS fraction. The addition of such particles improved their thermal stability, crystallinity, and gas barrier properties. Silver also conferred antimicrobial properties to the films. BM-containing samples exhibited strong antimicrobial activity against *Staphylococcus aureus*, *Escherichia coli*, and *Salmonella typhimurium bacteria*. All these characteristics and the used starting materials define a sustainable strategy to fabricate functional food packaging films.

In their review, Sarika et al. describe the main bio-based alternatives in the production of phenol and formaldehyde (PF) resins [3]. They introduce the topic highlighting the uses of PF resins and main reasons to substitute such substances: non-renewability of petroleum, problems related to health and the safety of phenol and formaldehyde, and the enhancement of specific features of PF resins. Lignins, tannins, and cardanol are shown to be the most important substitutes of phenol, while for formaldehyde, the best alternatives are hydroxymethylfurfural, furfural, and glyoxal. Finally, some technical and economic aspects of the bio-based PF resins are discussed.

Reichert et al. reviewed the state of the art, including materials, modifications, industrial uses, and different sustainability aspects, of bio-based packaging and textile materials [4]. In particular, polylactide (PLA), polyethylene furanoate, PBS, polyhydroxyalkanoates, cellulose, starch, proteins, lipids, and waxes are described and compared. Material modification is summarized in terms of biocomposites, fillers, and additives, as well as the different processing methodologies for bio-based packaging and textile polymeric materials. The main bio-based items (i.e., films, trays, pouches, bags, textiles, and nets) produced on a large scale are also reviewed. To conclude, the factors influencing sustainability, biodegradation, and recycling are also shown and discussed.

Martin et al. evaluated the influence of rapeseed press cake (RPC, a protein and fiber-rich by-product of oil pressing), extrusion temperature and moisture, and starch type (i.e., native and waxy) on the rheology, expansion properties, specific hardness, water binding and solubility, and extruder response of extruded starch/RPC blends [5]. The authors showed that higher proportions of RPC increased the complex viscosity of the corresponding blends. This was associated with a specific expansion behavior indicative of a pronounced sectional and decreased longitudinal expansion of the blends. 

Quiles et al. investigated the formation of aesthetically attractive and mechanically robust PBS-based biocomposites by adding a commercially available red pigment, itaconic acid (IA), to improve color fixation and ZrO_2_ nanoparticles to improve the mechanical properties [6]. IA allowed a good fixation of the pigment, although the samples were yellowish and the gloss was affected due to the optical properties of the dicarboxylic acid. Moreover, IA’s presence produced an important aging effect as a consequence of a higher hydrolysis degradation induced by its hydrophilic character. On the other hand, the incorporation of zirconia nanoparticles at low concentrations increased the surface hardness with no evident modifications of other properties.

Kang et al. described a facile fabrication of biodegradable and biocompatible cross-linked gelatin as screen printing substrates [7]. The authors modified the gelatin by cross-linking at different degrees. The sample with the highest degree of cross-linking showed lower swelling ratio and better mechanical performances than those with lower or no cross-linking. In addition, the adhesion to different inks was evaluated, with the finding that the adhesion of silver ink on gelatin surface was higher than that for carbon ink. Moreover, the gelatin with the highest degree of cross-linking showed an electrical response similar to that of commercial products. This sample was also completely biocompatible.

Benítez et al. fabricated bio-based coatings for food metal packaging inspired by the plant biopolyester cutin as a green alternative to bisphenol A-based lacquers. In particular, 2–3 µm thick layers of 9,10,16-trihydroxyhexadecanoic (or aleuritic) acid were deposited on aluminum (Al), electrochemically tin-plated steel (ETP), and chromium-coated tin-free steel (TFS) substrates and polymerized by melt polycondensation [8]. The kinetics of the self-esterification revealed an important catalyst role of the metal surface, especially relevant for the tin oxide layer on ETP. The presence of oxygen from the air allowed an oxidative diol cleavage and additional re-esterification side reaction that increased the effective number of ester groups and resulted in better performances such as adhesion to the substrate, glossy appearance, and hydrophobicity.

Aldas et al. studied the interaction between gum rosin and gum rosin derivatives with Mater-Bi [9]. The samples were prepared by melt extrusion and further injection molding. A microscopic analysis revealed that the main phases (i.e., polybutylene adipate-*co*-terephthalate and amorphous and semicrystalline thermoplastic starch) of commercial Mater-Bi present a low miscibility. The addition of gum rosins and derivatives increased the solubility among the different components, regulating different parameters such as mechanical properties, oxygen permeability, and wettability.

Correa-Pacheco et al. prepared bio-based cinnamon essential oil-containing PLA/PBAT fibers by melt mixing of both polymers and subsequent extrusion where the essential oil was incorporated [10]. The addition of PBAT to PLA generated a two-phase system and ductile blends were obtained. The cinnamon essential oil acted as a plasticizer. The hydrolysis of these samples at physiological conditions was evaluated for 12 weeks. Low weight losses (<5%) were determined in this time. The authors propose the application of these blends in food preservation of horticultural products.

Sangregorio et al. presented a work about the cross-linking kinetics of humins by chemorheological analysis and model-free kinetics under isothermal and non-isothermal curing [11]. Both non-catalyzed and acid-catalyzed (i.e., by adding small amounts of *p*-toluenesulfonic acid) curing processes were analyzed. Humins auto self-cross-linked with temperature, although the reaction was faster with lower values of activation energy in the presence of the acid. The authors demonstrated that the curing of humins is a complex process where multiple reactions occur.

Tarrés et al. determined the intrinsic flexural strength and coupling factor of natural fiber reinforcement in PLA with 15 to 30% of bleached kraft softwood fibers [12]. The flexural strength was increased with the fiber content. On the other hand, the deformation of such composites was slightly decreased when the fiber content was higher due to the high stiffness of the PLA matrix. The fiber flexural strength factor (FFSF) was higher than the fiber tensile strength factor (FTSF). FFSF was similar to other pine fiber-reinforced composites, while FTSF was interestingly higher.

Since there are still many possibilities for the use of bioplastics as alternatives of common petroleum-based polymers, the topic of “Sustainable Bio-Based Polymers: Towards a Circular Bioeconomy” continues to be relevant, and a second part as a new Special Issue has been prepared for *Polymers*.
